# Computed tomographic evaluation of three types of screw trajectories for posterior cervical spine fixation: Cervical pedicle screw, lateral mass screw, and paravertebral foramen screw

**DOI:** 10.1097/MD.0000000000029857

**Published:** 2022-07-15

**Authors:** Keiichi Tsuda, Atsushi Tagami, Shuta Yamada, Kazuaki Yokota, Ko Chiba, Akihiko Yonekura, Masato Tomita, Makoto Osaki

**Affiliations:** a Department of Orthopedic Surgery, Nagasaki University Graduate School of Biomedical Sciences, Nagasaki, Japan

**Keywords:** cervical pedicle screw, CT attenuation values, lateral mass screw, paravertebral foramen screw

## Abstract

Computed tomography (CT) attenuation values of cervical spine were evaluated in vivo using a clinically relevant group.

To compare CT attenuation values between cervical pedicle screw (CPS), lateral mass screw (LMS), and paravertebral foramen screw (PVFS) trajectories.

CPS and LMS are commonly used for posterior fixation of the cervical spine. The PVFS method has been reported as a new method. CT attenuation values along the screw trajectory are reportedly associated with screw stability.

We identified 45 patients who had undergone whole-body CT for trauma with no injury to the cervical spine. Regions of interest (ROIs) were designated along the trajectories that would be used for CPS, LMS, and PVFS through vertebral pedicles and lateral masses of the C3–C6 vertebrae. CT attenuation values of each ROI were measured and compared between each screw trajectories at each cervical vertebral level. Participants were divided into Group I (age, 20–39 years; n = 12), Group II (age, 40–59 years; n = 17), and Group III (age, 60–79 years; n = 16). CT attenuation values of ROIs were compared between each age group.

PVFS trajectories showed higher CT attenuation values than LMS trajectories at every vertebral level and also higher values than CPS trajectories at C5 and C6 levels. CT attenuation values at C3 were lower than those at C4 in the LMS trajectory and lower than those at C5 and C6 in the PVFS trajectory. CT attenuation values were lower in the elder group (>60 years old) than in the other 2 groups for all screw trajectories.

CT attenuation values suggested that the PVFS technique may be useful for posterior fixation of the cervical spine in elder patients who require more secure fixation.

## 1. Introduction

Opportunities for the use of cervical spine instrumentation are increasing with the extension of healthy longevity and advances in medical technology. Cervical screws offer better fixation than wiring or hook systems, and are often used for posterior fixation of the middle and lower cervical spine. The major methods of screw insertion for posterior cervical spine fixation are the cervical pedicle screw (CPS)^[1,2]^ method and lateral mass screw (LMS)^[3]^ method. Because the CPS is inserted into the vertebral pedicle, which comprises thick cortical bone, the CPS method provide stronger fixation and pullout strength than the LMS method.^[4]^ However, the CPS method also runs the risk of damage to the vertebral artery and spinal nerve.^[5–7]^ In addition, the CPS cannot be used in patients for whom the diameter of the pedicle is less than the width of the screw.^[8]^ The LMS method offers the choice of using bi- or monocortical screws,^[9]^ the latter of which shows a lower risk of vascular or nerve damage compared with the CPS method.^[10,11]^ However, fractures of the lateral mass sometimes occur during LMS insertion.^[12,13]^ In addition, screw stability is generally difficult to obtain in patients with osteoporosis.^[14]^

Maki et al^[15]^ recently reported the paravertebral foramen screw (PVFS) method as a new technique for posterior cervical spine fixation. In the PVFS method, the screw is inserted to the cortical bone of the spinal canal. This technique is considered intermediate between the other 2 techniques, offering safer insertion than the CPS method and better fixation than the LMS method. The pullout strength of screws inserted in PVFS fixation is greater than that of screws inserted in LMS fixation, even in cases where the PVFS method was applied as salvage after fracture of the lateral mass.^[15]^ The PVFS method carries a lower risk of vascular injury because the screw does not reach the transverse foramen containing the vertebral artery. Another advantage is that rod connection is easier with PVFS than with LMS or CPS.

The strength of screw fixation is generally evaluated by measuring bone mineral density (BMD) or computed tomography (CT) attenuation values along the screw trajectory. Ishikawa et al^[16]^ measured volumetric BMD around the pedicle screw (PS-vBMD) of the lumbar spine by quantitative CT (QCT), and reported that PS-vBMD correlated positively with the insertion torque of the pedicle screw. Since the QCT method is not generally used for the cervical spine, no reports appear to have evaluated BMD or CT attenuation values of screw trajectories for cervical posterior fixation.

The objective of this study was to investigate the CT attenuation values of screw trajectories used in CPS, LMS, and PVFS fixation involving the cervical spine in healthy individuals.

## 2. Materials and Methods

### 2.1. Subjects

Forty-five patients (22 males, 23 females) who visited the Emergency Center of Nagasaki University Hospital with severe trauma between 2011 and 2013 and underwent CT from the cervical spine to the pelvis were included in the study. Patients with compression myelopathy, ossification of the posterior longitudinal ligament, cervical spinal injury, or previous metabolic bone disease and those under 20 years old were excluded. The mean age of patients was 50.6 ± 16.2 years (range, 20–79 years). Mean height was 161.9 ± 9.1 cm (range, 140–184.5 cm), mean weight was 60.1 ± 13.7 kg (range, 42.6–98 kg), and mean body mass index was 22.8 ± 4.4 kg/m^2^ (range, 16.3–40.8 kg/m^2^).

This study was conducted in compliance with the Declaration of Helsinki and the Ethical Guidelines for Medical and Health Research Involving Human Subjects, and was approved by the Nagasaki University Hospital Clinical Research Ethics Committee (approval no. 19061733).

### 2.2. CT protocol

CT from the cervical spine to the pelvis was performed using a 64-row multidetector row CT system (Aquilion 64; CANON, Tokyo, Japan). Imaging conditions were: tube voltage, 120 kV; tube current, 250 mA; collimator, 0.5 mm; reconstruction slice thickness, 1 mm; slice interval, 1 mm; field of view, 220 mm; matrix, 512 × 512; and Kernel FC80.

### 2.3. CT attenuation value measurement

**Figure 1.**
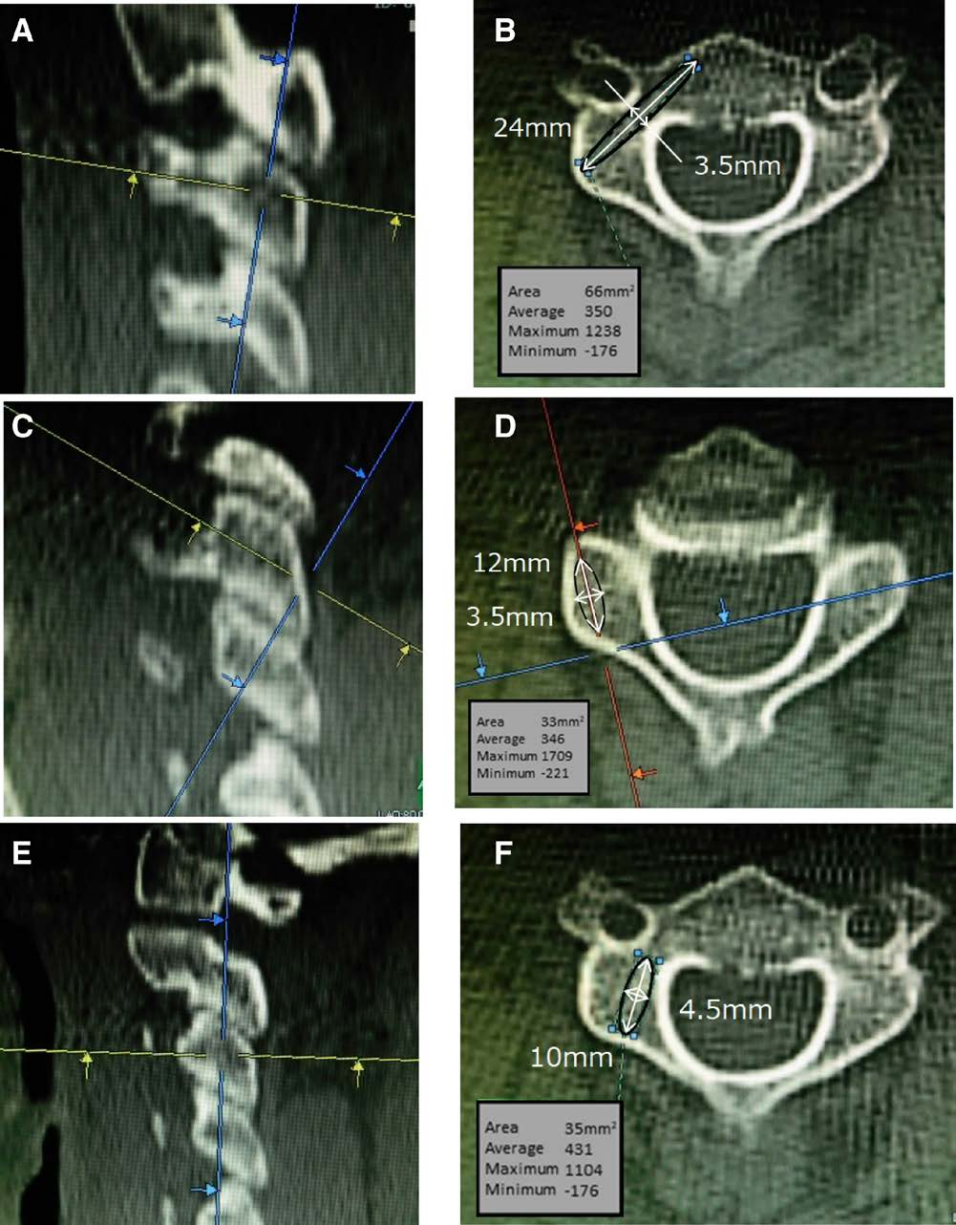
CPS trajectory: Oblique axial images aligned with the vertebral pedicle axis in sagittal images are created (A). The ROI is designated as an ellipse with a long diameter of 24 mm and a short diameter of 3.5 mm (B). LMS trajectory: Oblique axial images parallel to the facet joint in sagittal images are created (C). The ROI is designated as an ellipse with a long diameter of 12 mm and a short diameter of 3.5 mm (D). PVFS trajectory: Oblique axial images aligned with the vertebral pedicle axis in sagittal images are created (E). The ROI is designated as an ellipse with a long diameter of 10 mm and a short diameter of 4.5 mm (F). CPS = cervical pedicle screw, LMS = lateral mass screw, PVFS = paravertebral foramen screw, ROI = region of interest.

Multiplanar reconstruction (MPR) images of the simulated screw trajectories for the CPS, LMS, and PVFS methods were produced from acquired image data (Fig. 1) (Synapse Vincent, Fujifilm, Tokyo, Japan).

The insertion position of the CPS was selected to be along the pedicle axis of each vertebral body. Oblique cross-sectional images aligned with the vertebral pedicle axis based on screw inclination were therefore created (Fig. 1A). The region of interest (ROI) was designated as an ellipse with a long diameter of 24 mm and a short diameter of 3.5 mm following the trajectory of this oblique cross-sectional image (Fig. 1B).

Various methods for determining the position of LMS insertion have been reported.^[3,17–19]^ In this study, the trajectory was measured according to the Magerl method.^[3]^ In the Magerl method, the insertion point is 2 mm inside from the center of the lateral mass in the transverse section, and the screw is tilted outward at an angle of 25°. Oblique cross-sectional images were thus created based on the inclination of the LMS (Fig. 1C). The ROI was designated as an ellipse with a long diameter of 12 mm and a short diameter of 3.5 mm following the trajectory of this oblique cross-sectional image (Fig. 1D).

In the PVFS method, the insertion point is 1 mm inside from the center of the outer mass in the transverse section, and the screw is tilted inward at an angle of 20°. Oblique cross-sectional images were created based on the inclination of this screw (Fig. 1E). The ROI was designated as an ellipse with a long diameter of 10 mm and a short diameter of 4.5 mm following the trajectory of this oblique cross-sectional image (Fig. 1F).

CT attenuation values were measured in each ROI for each screw on both sides of the C3–C6 vertebrae in each patient.

To investigate age-related changes in CT attenuation values, patients were divided into 3 groups: Group I, 20–39 years old (n = 12); Group II, 40–59 years old (n = 17); and Group III, 60–79 years old (n = 16). Mean CT attenuation values on both sides of the C3–C6 vertebrae were calculated and compared in each group.

The CT attenuation value is a physical number that measures the X-ray absorption of a substance within a small unit volume for the subject of CT examination. CT attenuation values are expressed in Hounsfield units (HU).

### 2.4. Statistical analysis

Differences in CT attenuation values between different screw trajectories, different cervical vertebral levels, and different age groups were compared using the Steel-Dwass method. Statistical analysis was performed using JMP pro13 software (SAS Institute Japan, Tokyo, Japan), with values of *P* < .05 considered significant.

## 3. Results

**Figure 2.**
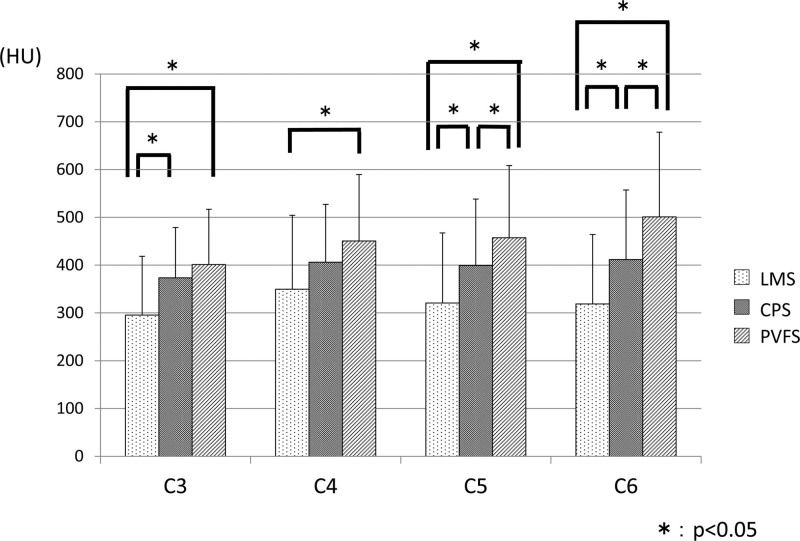
CT attenuation values are higher for the PVFS trajectory than for the LMS trajectory at every vertebral level and for the CPS trajectory at C5 and C6. CT = computed tomography, CPS = cervical pedicle screw, LMS = lateral mass screw, PVFS = paravertebral foramen screw.

In the LMS trajectory, CT attenuation values were statistically lower at C3 than at C4. Mean CT attenuation values in ROIs for the CPS trajectory ranged from 373 HU to 412 HU. In the PVFS trajectory, CT attenuation values at C3 were statistically lower than those at C5 or C6 (Table 1). CT attenuation values were lower for LMS trajectories than for CPS trajectories at C3, C5, and C6. CT attenuation values for the PVFS trajectory were higher than those for the LMS trajectory at every vertebral level and also higher than for the CPS trajectory at C5 and C6 (Fig. 2).

**Table 1:** CT attenuation values (in Hounsfield units) for the CPS, LMS, and PVFS trajectories in C3, C4, C5, and C6 vertebrae.

	LMS	CPS	PVFS
C3	296 ± 106	373 ± 124*	401 ± 116*
C4	349 ± 122‡	406 ± 156	451 ± 140*
C5	321 ± 140	400 ± 147*	457 ± 152*†‡
C6	319 ± 146	412 ± 146*	501 ± 178*†‡

The values are given as the mean and standard deviation.

CPS = cervical pedicle screw, LMS = lateral mass screw, PVFS = paravertebral foramen screw.

* Versus LMS, *P* < .05.

† Versus CPS, *P* < .05.

‡ Versus C3, *P* < .05.

Mean CT attenuation values in ROIs for LMS trajectories were 437 HU in Group I, 322 HU in Group II, and 234 HU in Group III. Mean CT attenuation values in ROIs for CPS trajectories were 491 HU in Group I, 419 HU in Group II, and 304 HU in Group III. Mean CT attenuation values in ROIs for PVFS trajectories were 535 HU in Group I, 474 HU in Group II, and 368 HU in Group III. CT attenuation values of all screw trajectories decreased with age and were lowest in Group III, the oldest patients (Table 2).

**Table 2:** CT attenuation values (in Hounsfield units) for the CPS, LMS, and PVFS trajectories in Groups I, II, and III.

	LMS	CPS	PVFS
Group I (20–39 y, n = 12)	437 ± 129*†	491 ± 140*†	535 ± 125*†
Group II (40–59 y, n = 17)	322 ± 101†	419 ± 131†	474 ± 155†
Group III (60–79 y, n = 16)	234 ± 82	304 ± 96	368 ± 122

The values are given as the mean and standard deviation.

CPS = cervical pedicle screw, LMS = lateral mass screw, PVFS = paravertebral foramen screw.

* Versus Group II, *P* < .05.

† Versus Group III, *P* < .05.

## 4. Discussion

Stability is crucial when performing cervical screw fixation of the spine, particularly in elderly patients. BMD is one factor that can be used to predict fixation strength. Overall BMD is usually evaluated by means of digital X-ray absorptiometry of the lumbar spine and proximal femur, and evaluation of local bone such as the vertebral pedicles and lateral masses of the cervical spine is not feasible. Local BMD can be evaluated by QCT. No previous reports appear to have examined BMD or CT attenuation values for the trajectories of different screws in the cervical spine.

The present results showed that CT attenuation values for the CPS trajectory at C3–C6 were higher than those for the LMS trajectory. Jones et al^[4]^ inserted CPS and LMS into cadaveric vertebral bodies and measured pullout strengths, finding that CPS provided higher pullout strength and stronger fixation than LMS. Hostin et al^[20]^ revised a model of LMS insertion and removal in cadaveric vertebral bodies, and measured insertion torque and pullout strength of LMS reinsertion and CPS insertion. They reported that CPS provided greater pullout strength than LMS. They also showed that higher BMD resulted in greater insertion torque and pullout strength.^[20]^ Schreiber et al^[21]^ reported that CT attenuation values obtained from clinical CT performed for other purposes correlate with BMD. Higher CT attenuation values for CPS trajectory than for LMS are likely to represent higher local BMD and may be associated with the greater stability of the CPS method compared with the LMS method.

**Figure 3.**
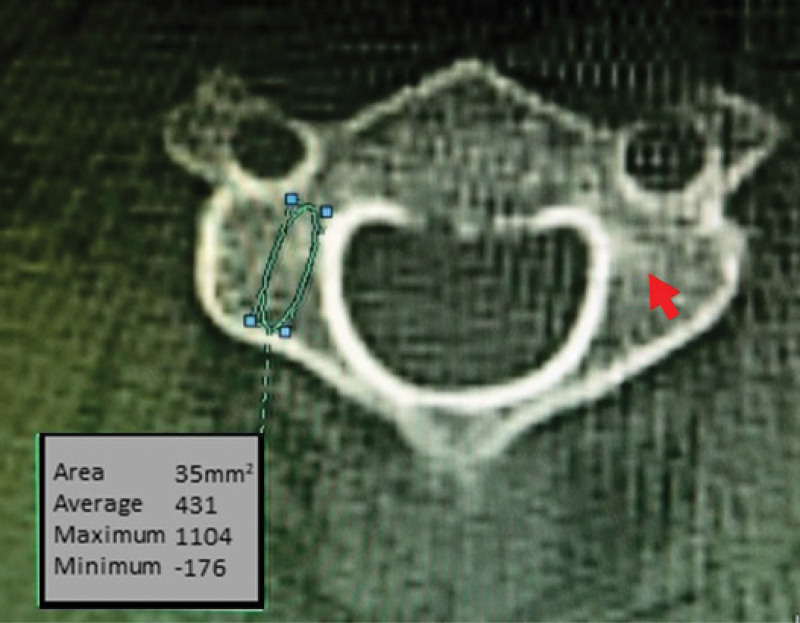
High-density areas are often observed in the PVFS trajectory. PVFS = paravertebral foramen screw.

Comparing CT attenuation values between CPS, LMS, and PVFS trajectories, CT attenuation values were highest for the PVFS trajectory and lowest for the LMS trajectory. Pullout strength may thus be greater for PVFS than for CPS or LMS. CT attenuation values of the lumbar vertebrae have been reported as predictors of pedicle screw loosening after lumbar fusion surgery.^[22]^ Matsukawa et al^[23]^ reported higher screw insertion torque in lumbar fusion as the regional CT attenuation value increased, and noted that regional CT attenuation was more predictive of insertional torque than overall BMD. Higher CT attenuation values in ROIs in the C3–C6 vertebrae of the middle and lower cervical spine may also correlate with higher screw pullout strength. Anderst et al^[24]^ reported that the BMD of the vertebral pedicles of the cervical spine is greater than that of the laminae and lateral masses. Our study found many areas of osteosclerosis at the leading tip of the PVFS trajectory on CT (Fig. 3). These osteosclerotic areas were only found in the PVFS trajectory, not in the CPS or LMS trajectory. In the PVFS trajectory, osteosclerotic areas were found in 21 of 45 cases at C3, 33 cases at C4, 36 cases at C5, and 35 cases at C6. These high-density areas at the screw tip may contribute to higher CT attenuation values for the PVFS trajectory.

CT attenuation values for the LMS trajectory were higher in C4 than in C3. CT attenuation values for the PVFS trajectory were also higher in C5 and C6 than in C3. Degenerative changes in the cervical spine tend to be more common at lower levels, and osteoarthritic changes are more likely at the facet joints. In cancellous bone close to sites of such osteoarthritic changes, the trabecular structure thickens and BMD also increases.^[25]^ This suggests that degenerative changes in the cervical spine may be one factors contributing to higher CT attenuation values of the lower cervical spine.

In all screw trajectories, CT attenuation values tended to decrease with age. Pickhardt et al^[26]^ measured CT attenuation values in the thoracolumbar vertebrae of patients with osteoporosis or osteopenia, and in healthy individuals. They reported that values were lowest in patients with osteoporosis.^[26]^ Because BMD tends to decrease with age, CT attenuation values may show a similar trend. Our results also indicate that older patients required greater screw fixation strength in the cervical spine.

This study had a number of limitations that should be kept in mind when interpreting the results. First, we evaluated only CT attenuation values, not BMD using QCT methods. In this study, all CT was performed using the same CT device under the same clinical conditions in an attempt to eliminate variation errors in CT attenuation values. Second, many additional factors that may affect screw fixation were not addressed. For example, the length and diameter of screws varies between cases. This study cannot clarify the pullout strength or insertional torque of each screw. Third, we used two-dimensional evaluations, not three-dimensional evaluations that match the shapes of screws. Last, the number of subjects was relatively small. However, this method is easily applied to clinical settings, and we consider that it has potential for use in daily clinical practice.

CT attenuation values are simple to measure in any arbitrary ROI, and may prove helpful for choosing cervical screws and predicting screw stability. CT evaluation is thus useful in the performance of cervical spine fixation.

## 5. Conclusions

CT attenuation values for LMS, CPS, and PVFS trajectories were measured. CT attenuation values were higher for the PVFS trajectory than for the LMS trajectory at every vertebral level and for the CPS trajectory at C5 and C6. CT attenuation values were lowest in the elderly group for all screw trajectories. CT attenuation values may be helpful in choosing cervical screws and predicting screw stability in daily clinical practice. These findings suggest PVFS as a potentially useful technique for posterior fixation of the cervical spine in elderly patients who require more secure fixation.

## Acknowledgments

We would like to thank Shuntaro Sato, a specialist in biostatistics, for providing advice on the statistical analyses.

## Author contributions

**Conceptualization:** Keiichi Tsuda, Atsushi Tagami, Shuta Yamada, Kazuaki Yokota, Makoto Osaki

**Data curation:** Keiichi Tsuda, Atsushi Tagami, Kazuaki Yokota, Ko Chiba, Makoto Osaki

**Formal analysis:** Keiichi Tsuda, Atsushi Tagami, Kazuaki Yokota, Ko Chiba, Makoto Osaki

**Investigation:** Keiichi Tsuda, Atsushi Tagami, Shuta Yamada, Kazuaki Yokota, Ko Chiba, Makoto Osaki

**Methodology:** Keiichi Tsuda, Atsushi Tagami, Kazuaki Yokota, Ko Chiba, Makoto Osaki

**Project administration:** Keiichi Tsuda, Atsushi Tagami, Ko Chiba, Akihiko Yonekura, Masato Tomita, Makoto Osaki

**Resources:** Keiichi Tsuda, Atsushi Tagami, Shuta Yamada, Kazuaki Yokota, Makoto Osaki

**Software:** Keiichi Tsuda, Atsushi Tagami, Ko Chiba, Makoto Osaki

**Supervision:** Keiichi Tsuda, Atsushi Tagami, Kazuaki Yokota, Ko Chiba, Makoto Osaki

**Validation:** Keiichi Tsuda, Atsushi Tagami, Kazuaki Yokota, Ko Chiba, Makoto Osaki

**Visualization:** Keiichi Tsuda, Atsushi Tagami, Ko Chiba, Akihiko Yonekura, Masato Tomita, Makoto Osaki

**Writing – original draft:** Keiichi Tsuda, Atsushi Tagami, Ko Chiba, Makoto Osaki

**Writing – review & editing:** Keiichi Tsuda, Atsushi Tagami, Ko Chiba, Akihiko Yonekura, Masato Tomita, Makoto Osaki
